# Elbow Gesture Recognition with an Array of Inductive Sensors and Machine Learning

**DOI:** 10.3390/s24134202

**Published:** 2024-06-28

**Authors:** Alma Abbasnia, Maryam Ravan, Reza K. Amineh

**Affiliations:** Department of Electrical and Computer Engineering, New York Institute of Technology, New York, NY 10023, USA; aabbasni@nyit.edu (A.A.); mravan@nyit.edu (M.R.)

**Keywords:** gesture recognition, inductive sensors, machine learning

## Abstract

This work presents a novel approach for elbow gesture recognition using an array of inductive sensors and a machine learning algorithm (MLA). This paper describes the design of the inductive sensor array integrated into a flexible and wearable sleeve. The sensor array consists of coils sewn onto the sleeve, which form an LC tank circuit along with the externally connected inductors and capacitors. Changes in the elbow position modulate the inductance of these coils, allowing the sensor array to capture a range of elbow movements. The signal processing and random forest MLA to recognize 10 different elbow gestures are described. Rigorous evaluation on 8 subjects and data augmentation, which leveraged the dataset to 1270 trials per gesture, enabled the system to achieve remarkable accuracy of 98.3% and 98.5% using 5-fold cross-validation and leave-one-subject-out cross-validation, respectively. The test performance was then assessed using data collected from five new subjects. The high classification accuracy of 94% demonstrates the generalizability of the designed system. The proposed solution addresses the limitations of existing elbow gesture recognition designs and offers a practical and effective approach for intuitive human–machine interaction.

## 1. Introduction

The field of wearable technology has seen remarkable progress in recent years. Flexible materials, with their advantages like lightness, high stretchability, and user comfort, have enabled the development of devices that integrate seamlessly into our daily lives. This has led to the growing popularity of wearable sensing devices [1,2]. These sensors can be worn by users without significantly affecting their regular activities. Wearable sensors offer benefits such as small size, lightweight, and the ability to monitor biomedical parameters over long periods efficiently and comfortably. Wearable sensing technologies have found diverse applications, from soft robotics [3] and health tracking [4] to human–machine interfaces [5]. As the field continues to evolve, we can expect even more transformative solutions that leverage the unique capabilities of flexible materials.

A variety of wearable sensors, such as micro-electro-mechanical systems (MEMS), capacitive, electromyography (EMG), microfluidic triboelectric sensors (FMTS), strain, resistive, and inductive sensors, have been under thorough investigation for their potential in various applications for wearable devices. Each of these sensor types has its advantages and limitations. Here, we present a brief discussion of these sensing techniques, highlighting their unique features and considerations.

Advancements in sensor technology are driving the shift from rigid to flexible materials in wearable devices, improving comfort. Integrated circuits (ICs) and MEMS are key technologies enabling this transition by miniaturizing sensor chips. For instance, in [6], a human motion capture and recognition system using MEMS sensor nodes was developed to monitor human joint rehabilitation. The system uses MEMS sensor nodes and a two-stage extended Kalman filter algorithm for multi-sensor data fusion. A stationary posture calibration method is used to calculate the error matrix between the sensor node and body coordinate systems. However, MEMS sensors have limitations, including susceptibility to noise and the need for complex calibration procedures, which impact their accuracy and reliability in certain applications and make them inflexible.

Capacitive sensing operates on the principle of detecting changes in capacitance. These sensors can detect a wide range of gestures, from simple swipes and taps to more complex hand movements, making them versatile for various applications. For example, in [7], a non-contact capacitive sensing method was presented for recognizing forearm motions. The proposed capacitive sensing system records upper limb motion information from muscle contractions without direct skin contact. Also, in [8], a wearable capacitive sensing system was presented for recognizing lower limb locomotion modes, which includes sensing bands, a signal processing circuit, and a gait event detection module. One potential advantage of the non-contact capacitive sensing approach is that it avoids the direct skin contact required by other techniques, which could improve user comfort and ease of use. However, a potential limitation is that capacitive sensing can be more susceptible to external disturbances or environmental factors, which could impact the reliability and accuracy of the motion recognition.

Another common approach for detecting upper and lower limb movements is the use of EMG sensors. EMG can provide valuable information about muscle activity and movement intention. For instance, in [9], an algorithm was developed to detect the onset of upper limb reaching movements using surface electromyography (EMG) signals from multiple muscles to enable real-time control of upper limb exoskeletons. In [10], an upper limb prosthetic device was developed that can be controlled by myoelectric (EMG) signals. In [11], the authors investigated the use of surface electromyography (EMG) signals from hip muscles and residual limb muscles to detect changes in the locomotion mode for users of above-knee prosthetic limbs. While the key advantage of the EMG-based approach appears to be the ability to detect movement intention before the actual movement occurs, a potential limitation is that the system still requires surface EMG sensor placement, which may not be comfortable. Given that EMG signals are so weak, with amplitudes at the microvolt level, they are difficult to extract from noisy backgrounds [12,13]. As a result, complex processing approaches involving filtering, amplification, and other techniques are often required to obtain usable EMG signals, which increases the expense and complexity of the circuit design for EMG-based prosthetic systems.

In [14], researchers have introduced a pioneering method for hand gesture recognition using wearable ultrasound technology. There, a one-dimensional (1D) convolutional autoencoder was utilized to compress raw ultrasound data by a factor of 20 while preserving crucial amplitude features. These compressed data were then utilized to train an XGBoost classifier, achieving an impressive classification accuracy of 96%. This approach overcomes the limitations of traditional surface electromyography (sEMG) methods, which are restricted to monitoring superficial muscles and are susceptible to crosstalk between neighboring muscle fibers. However, the ultrasound method has its limitations, including imaging depth, resolution, field of view, and tissue penetration.

Another novel sensor used in human–machine interface applications is the flexible microfluidic triboelectric sensor (FMTS). In [15], researchers developed an FMTS designed to improve the reliability of self-powered human–machine interfaces in complex real-world environments. This sensor boasts a high transmittance of 82% and exhibits flexible, twistable, and conformable properties, making it ideal for skin attachment. By harnessing triboelectrification and electrostatic induction between a liquid stream, microchannel, and interdigital electrodes, the FMTS generates measurable voltage wave peaks.

Another widely utilized sensor in the realm of wearable technology is the strain sensor, which functions by altering its electrical resistance in response to mechanical deformation. In this context, the researchers in [16] developed a highly sensitive glove for monitoring finger motion, specifically designed for hand rehabilitation. This glove employs printed strain sensors on a rubber base with an intrinsic surface microstructure. These strain sensors, crafted from specialized ink and encapsulated with Ecoflex, demonstrated high strain sensitivity, a broad detection range, rapid response and recovery times, and impressive durability. The data collected by the glove were used to train a neural network model, allowing for accurate real-time monitoring and assessment of hand movements, thereby aiding in rehabilitation efforts. In [17], researchers developed an e-textile sleeve system to recognize arm gestures, aiming to overcome obstacles faced by computer vision techniques such as obfuscation and lighting conditions. The system integrates multiple ultra-sensitive graphene e-textile strain sensors along with an inertia measurement unit into a sports sleeve. The study includes the design and fabrication process of the sensors, as well as a detachable hardware implementation for reconfiguring the processing unit to other body parts. A user study with ten participants showed that the system could classify six different fundamental arm gestures with over 90% accuracy. However, the limitations of strain sensors, especially those based on e-textiles, can include sensitivity to environmental conditions, such as humidity and temperature, and limited durability over time.

As discussed above, the working principle of stretchable resistive strain sensors relies on the concept of mechanical strain [18], where physical deformation leads to changes in the electrical resistance. While these sensors can exhibit high sensitivity, their primary limitation is the direct relationship between mechanical deformation and electrical resistance. This means that even nominal operating heat can cause mechanical changes, which in turn affects the electrical resistance [19]. This characteristic makes these types of strain sensors challenging to use for extended durations, as the mechanical deformation can impact the sensor’s performance and reliability over time. They offer unique benefits but suffer specific challenges. In [20], the researchers developed a wearable gesture-sensing device for monitoring the flexion angles of the elbow and knee joints. The device incorporates a textile strain sensor made of elastic conductive webbing, which exhibits a linear relationship between its electrical resistance and the flexion angle. To calibrate the device, the researchers established an equation relating the flexion angle to the resistance measured by the textile sensor using a custom-built apparatus. However, the calibration process requires an assembled apparatus with a protractor, which may not be practical for all end-use scenarios where a quick setup is desirable. Another work using resistive sensors was presented in [21]. There, a smart textile sensor was developed based on the piezo-resistive effect, composed of a mix of conductive and dielectric threads arranged in a double bridle crochet structure, to monitor elbow flexion. This sensor was integrated into a sweater, and a deep neural network model was trained to accurately recognize the elbow joint angle from the sensor data.

Lastly, a wearable technology that has recently gained attention is the emerging inductive wearable sensors. These sensors are constructed using highly conductive threads [22,23,24], which enables the sensors to be easily incorporated into different materials and fabrics, expanding the potential applications and integration of sensing capabilities. Unlike commercially available inductive coils with specific shapes, such as rectangular or circular, sewing conductive threads allows for limitless size and shape possibilities. The geometric design parameters of these coils, including shape, size, gap between the turns, number of turns, and inner-to-outer diameter ratio, are crucial in maximizing sensor sensitivity. Therefore, finding the optimal design and their placement on the body is critical for enhancing the performance of inductive textile sensors [25,26,27]. Moreover, inductive sensors are highly sensitive and can detect subtle changes in the magnetic field or coil’s physical deformation, making them ideal for capturing detailed motion and physiological data. This level of sensitivity opens new possibilities for real-time monitoring and analysis, enabling better decision making and personalized interventions. Previous studies have shown that inductive textile sensors, utilizing conductive threads, are responsive to physical deformations, making them suitable for a variety of applications. In [28], a flexible magnetic induction-sensing device was developed that can be integrated into fabrics and attached to the human body to monitor changes in inductance during rehabilitation exercises (for arms and abdomen) and daily activities. This sensor system leverages specialized adhesives and conductive fibers to provide high sensitivity, reliability, and repeatability in detecting joint movements, offering a comfortable and non-invasive wearable solution for real-time and long-term monitoring applications. Another work that employed inductive sensors was reported in [29]. There, the performance of three different configurations of wearable inductive sensors for monitoring human joint movements was evaluated: a single planar rectangular coil, two separated planar coils connected in series, and two helical coils connected in series. Through simulations, they analyzed the sensitivity of these sensor designs in terms of the change in the resonant frequency of the tank circuits, including the sensor coils, when the elbow joint angle was varied.

In this study, we expanded our work to detect various elbow movements. We employed textile rectangular coils to detect a range of elbow gestures. To deliver accurate outcomes while being flexible and comfortable for the user to wear, conductive threads were sewn on patches and placed strategically on a compression arm sleeve in customizable sizes to capture complex elbow movements. To measure the sensors, we implemented a data-acquisition system, measuring a tank circuit formed by each sewn coil and external inductors and capacitors. The coils’ inductances changed with elbow gestures, subsequently altering the resonant frequency of the corresponding LC tank circuits. These variations were captured by the data-acquisition circuit and conveyed to the computer for processing. Employing a machine learning algorithm (MLA), the acquired responses were analyzed to recognize a large range of elbow movements.

## 2. Materials and Methods

**Figure 1:**
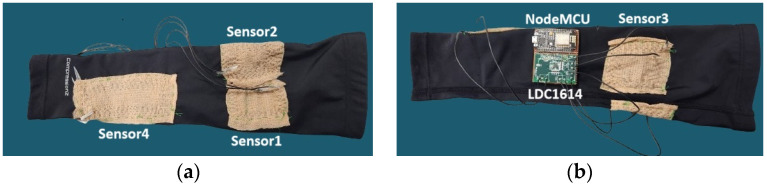
(**a**) Sleeve front side (**b**) Sleeve back side.

In this section, we introduce the operational concept of our proposed elbow gesture recognition system. Our objective is to develop a wearable sensor that prioritizes cost-effectiveness and user convenience. To achieve this, we employed an available conductive thread from Adafruit (the stainless conductive yarn with a resistance of 1 Ω/inch and a diameter of 0.4 mm [30]) to delicately stitch coils onto patches out of a commercial self-adherent wrap material. The patches were then sewn on a commercially accessible athletic arm sleeve, as shown in Figure 1. In this study, single-layer rectangular coil designs were integrated into four different positions on the arm and around the elbow joint to detect a wide range of elbow movements and complex gestures. The parameters of the four textile-based wearable inductive sensors presented in Figure 1 can be attributed to several key design considerations for the planar spiral coils, as discussed in [31], including the number of turns *n*, turn width *w*, turn spacing *s*, inner diameter *d_in_*, and outer diameter *d_out_*. The inner and outer diameters are typically used to define two other dependent parameters: the average diameter *d_avg_* = 0.5 (*d_out_* + *d_in_*) and the fill ratio *ρ* = (*d_out_* − *d_in_*)/(*d_out_* + *d_in_*). In [31], by slightly modifying Wheeler’s formula, they derived a valid expression for the inductance of the coils as *L* = (*k*_1_*µ*_0_*n*^2^*d_avg_*)/(1 + *k*_2_*ρ*), where *ρ* is the fill ratio defined earlier, and the coefficients *k*_1_ and *k*_2_ depend on the layout, which are 2.34 and 2.75, respectively, for the rectangular coils. The ratio *ρ* indicates how hollow the inductor is; a small *ρ* means a hollow inductor, while a large *ρ* indicates a full inductor. Two inductors with the same average diameter but different fill ratios will have different inductance values; the fuller inductor will have a lower inductance due to its inner turns, contributing less positive mutual inductance and more negative mutual inductance. Given this information and considering practical constraints for the elbow size, we chose different parameters for each coil placed at an optimal position on the elbow to capture the motions. Moreover, smaller spacing between turns enhances interwinding magnetic coupling and reduces the occupied area. Considering sewing limitations, we chose 2 mm gap between the turns. Overall, we experimented with different positions and parameters and selected the optimal design and placement for accurate and repeatable responses for our desired elbow gestures.

Table 1 demonstrates the final parameters employed in the single-layer rectangular coils for the four different positions on the arm and around the elbow joint, as shown in Figure 1. The primary aim in determining the optimal parameter of the coils was to maximize the change in the resonant frequency of the utilized LC tank circuit (described in the next paragraph) as the corresponding coil undergoes a transition from its initial non-deformed state (position 1) to its maximum deformation (position 2). This objective emphasizes the significance of detecting substantial frequency variations in response to the coils’ deformations.

**Figure 2:**
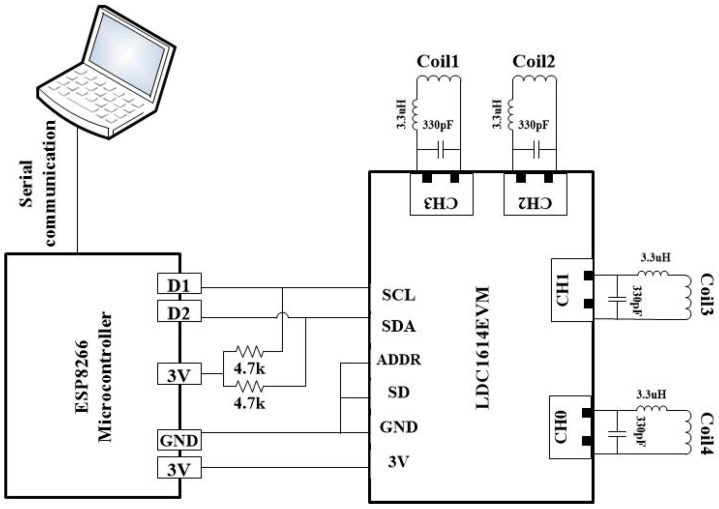
Block diagram of the gesture recognition system including four coils, inductance-to-digital converter (LDC1614EVM), microcontroller (NodeMCU Esp8266), and PC.

The sensing mechanism of the sewn inductive sensors is based on the measurement of the resonant frequency of an LC tank circuit. This circuit comprises the meticulously stitched coil’s inductance on the glove, which is connected in series with an external inductor of 3.3 µH. The series coils are connected in parallel to an external capacitor of 330 pF. To facilitate the execution of the data-acquisition system, we have implemented the board depicted in Figure 1. This system comprises two main components: the LDC1614EVM [32] and a NodeMCU Esp8266 developed by Espressif, Shanghai, China [33]. The LDC1614EVM, developed by Texas Instruments, Dallas, Texas, USA is an evaluation board for a 4-channel 28-bit inductance to digital converter (LDC). The NodeMCU Esp8266 acts as the interface for transmitting data from the system to a laptop via a USB cable. The acquired data are further processed using MATLAB R2022a. Figure 2 shows the hardware components of the system.

As discussed, the measurement of four sensors is accomplished using the LDC1614EVM board, while the NodeMCU Esp8266 microcontroller retrieves data from the LDC board using the I^2^C protocol. Subsequently, the microcontroller transfers the acquired data to the laptop via a USB cable and serial communication. This integrated approach ensures efficient and comprehensive data acquisition for the sensor system. Notably, to minimize the size of the LDC board, unnecessary factory-installed components, such as the USB port and a microcontroller unit, were removed. As a result of this modification, two pins, namely, SCL and SDA, were connected to the corresponding pins on the NodeMCU Esp8266 microcontroller using 4.7 kΩ pull-up resistors to achieve a high level of logic. Additionally, the ADDR and SD pins were connected to the ground to prevent floating.

Any physical deformation that alters the initial shape of the sewn coil on the sleeve results in changes in the total inductance and, consequently, the resonant frequency of the tank circuit. This shift in the resonant frequency serves as an indicator for monitoring elbow gestures [25].

**Figure 3:**
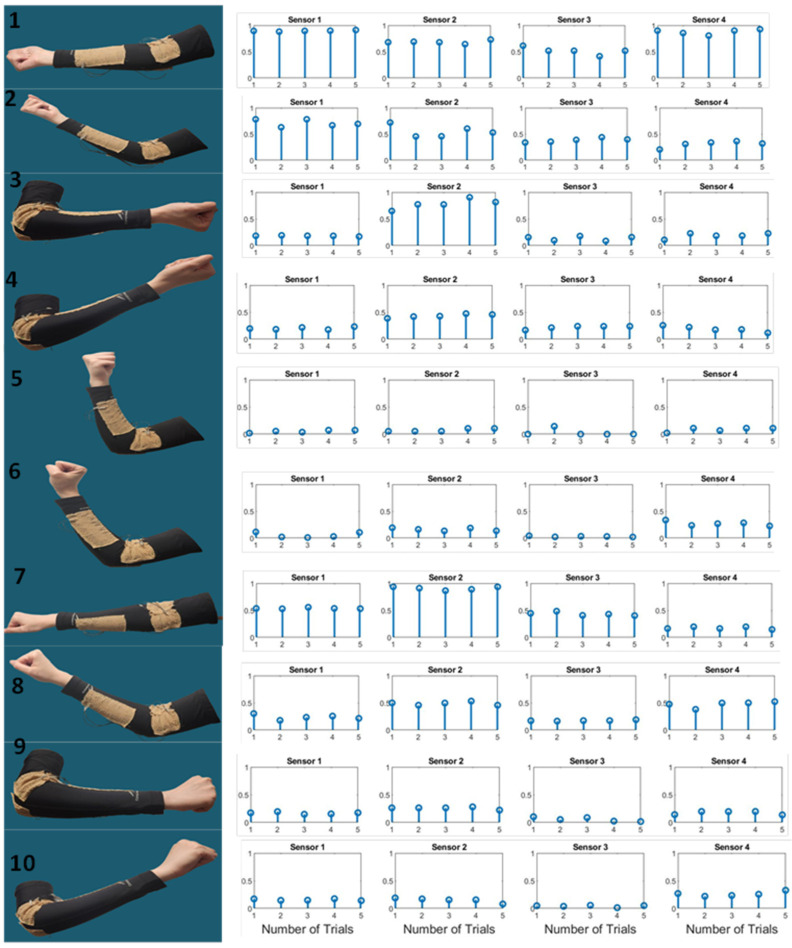
Resonant frequency alterations corresponding to 10 elbow gestures for a user and for 5 repetitions. The *y*-axis corresponds to the normalized resonant frequency alterations, with values ranging between 0 and 1, and the *x*-axis represents the number of trials. Each of the 5 trials corresponds to 1 repetition of the elbow gesture, indicating that we conducted 5 repetitions for each elbow gesture.

For elbow gesture recognition, we considered 10 gestures, as shown in Figure 3. These gestures can be divided into two sets: gestures 1–5 represent basic elbow flexion and extension movements, while gestures 6–10 are similar but with an added component of hand twisting. This distinction allows us to investigate the impact of hand orientation, in addition to elbow joint kinematics, on the observed resonant frequency patterns. These 10 gestures are visually depicted, providing a clear representation of the various elbow movements and positions that were examined. Furthermore, Figure 3 shows the corresponding resonant frequency alterations observed for each of these 10 gestures, with the *y*-axis corresponding to the normalized resonant frequency alterations, with values ranging between 0 and 1 and the *x*-axis representing the number of trials. Each of the five trials corresponds to one repetition of the elbow gesture, indicating that we conducted five repetitions for each elbow gesture. Specifically, the resonant frequency changes are presented for one individual participant, across five repetitions of each gesture, and for four different sensors. In this figure, we demonstrate the consistency and reliability of their measurements. We can observe the unique resonant frequency patterns associated with different elbow gestures. Table 2 presents the frequency variations observed for 4 sensors and the 10 gestures shown earlier in Figure 3. Regarding the variation in resonant frequency alterations among different subjects, data provided in Table 3, which are the resonant frequency alternations (mean ± std) in MHz for each sensor for all gestures among all participants, suggest that the variation among all participants is greater than within a single participant. We believe that this is due to the differences in how the individuals perform the gestures and the anatomy of their elbows.

The sensors respond to shape deformations, which are combinations of bending and stretching. Thus, Table 4 presents the sensitivity of the sensors in three distinct postures: front bending, side bending, and twisting. Sensitivity is defined as the ratio of the change in resonant frequency over the change in the angle from the resting position for each gesture. The system’s response range can vary across individuals (as shown in Table 3). Thus, here, we provide sensitivity values based on the responses obtained from one individual.

The proposed system demonstrated a fast response time of 50 ms, mainly determined by the speed of data acquisition and transmission by the microcontroller.

## 3. Data Collection and Processing

This study’s initial phase involved data collection as individuals gradually transitioned their hands from a resting to a gesture position. In this study, the resting position is defined as maintaining the elbow joint in a 90-degree bent position, akin to gesture 5 in Figure 3. Each measurement cycle starts with participants holding the resting position for five samples, followed by a beep sound prompting the user to start a new gesture, which is then held for another five samples. This cycle repeats 5 times per participant, resulting in 50 samples per individual. The responses of four coils are measured at 200 ms intervals, and the data are transmitted to the laptop via a USB cable. As we showed earlier in Figure 3, all sensors exhibit measurable, consistent, and unique response patterns to the 10 targeted gestures.

The research enlisted the participation of 13 healthy individuals aged 20–30 in accordance with the Institutional Review Board of the New York Institute of Technology’s policies. Each participant wore the sleeve and performed 10 gestures, repeated 5 times each, resulting in 50 trials per gesture. For elbow gesture detection, variations in the resonant frequencies were analyzed using MLAs, involving a training and validation phase and a testing phase. Eight subjects were included in the training and validation phase, and the remaining five subjects were included in the testing phase. During training, features extracted from 4 coils and their labels for the 10 gestures were used. The changes in resonant frequencies caused by each gesture were concatenated into a vector and used for MLA training. To pre-process sensor data, a two-step approach was employed. Firstly, data for each gesture were calibrated by subtracting the mean of the initial 5 samples corresponding to the resting position from the subsequent 10 samples. Then, the energy of samples corresponding to each gesture was computed. The energy of all sensors was concatenated into a feature vector for each gesture. To improve machine learning performance and address low accuracy with leave-one-subject-out cross-validation (LOSO-CV), a data-augmentation strategy was adopted. This involved calculating the mean of trials from different subjects for each gesture and sensor, creating new datasets while maintaining original data characteristics. Various combinations of subjects were used to generate new datasets, significantly expanding the dataset to include 246 new datasets (1230 new sets of trials). Incorporating the data-augmentation strategy into our study offers several notable advantages. Firstly, by diversifying our dataset, we can improve the model’s ability to generalize, reducing the risk of overfitting and enhancing its performance on new, unseen data—critical for real-world applications. Secondly, the augmented dataset allows the model to learn more deeply about the nuances of elbow gestures, potentially leading to higher classification accuracy. Moreover, this approach helps mitigate biases that might arise from individual subject characteristics, thereby bolstering the robustness of our model. By thoughtfully selecting subjects and generating diverse combinations, we can avoid dataset bias, ensuring that our model’s performance is not skewed by specific subject traits.

The MLA was trained using the selected features from the four coils, along with corresponding gesture labels. To evaluate the MLA’s performance and the device’s functionality, a dataset consisting of 1270 trials for each gesture was collected, comprising 1230 augmented trials and 40 trials taken from 8 participants. Gesture recognition accuracy was initially validated using a 5-fold cross-validation (5F-CV) approach, which randomly divides the dataset into 5 folds, using 20% of trials for testing and 80% for training in each fold. This process is repeated 20 times with different random splits of the data to ensure result reliability. The average classification performance across all 20 runs is then calculated, providing a robust evaluation of the MLA’s accuracy and the system’s reliability. To facilitate cross-validation for our statistical model validation, we utilized the cvpartition function in MATLAB R2022a. This function enabled us to create a randomized partition on our dataset, which is crucial for robust cross-validation. Specifically, we configured the function with the parameters necessary for our study, such as the number of folds (5 in this case) and the stratification setting to ensure balanced class proportions across folds. By stratifying the data in this manner, we aimed to prevent potential biases stemming from imbalanced class distributions, thus improving the accuracy and generalizability of our model. Overall, this approach allowed us to effectively validate our model’s performance across different folds, enhancing the reliability of our statistical analyses.

To further evaluate the model’s generalizability and the system’s robustness, we employed LOSO-CV, using data from 253 subjects for training in each iteration while reserving data from one subject for testing. This method ensures the MLA is evaluated on unseen data from subjects, offering a comprehensive assessment of its performance across a diverse range of individuals. LOSO-CV is particularly valuable in evaluating how well the MLA can generalize to new subjects, a crucial factor in applications such as gesture recognition, where individual characteristics can significantly impact performance. Its use adds a layer of validation to the study, demonstrating the model’s consistent performance across different subjects and highlighting the sleeve’s robustness.

For classification, we employed the random forest (RF) classifier, utilizing the functionalities of the Statistics and Machine Learning Toolbox in MATLAB R2022a. RF is an ensemble MLA consisting of multiple decision tree (DT) classifiers that collaborate to predict a class for a new data sample. Each DT in the ensemble independently predicts a class, and the class with the highest number of votes across all DTs is selected as the final predicted class. This approach aggregates predictions from multiple DTs, enhancing the classifier’s generalization ability and robustness by reducing overfitting and increasing resilience to outliers. We developed RF using MATLAB’s Tree Bagger function, part of the Statistics and Machine Learning Toolbox. Our implementation involved creating a Tree Bagger object with 50 decision trees, using the training features (input variables) and their corresponding targets (labels).

We then tested the performance of the RF classifier by using a new dataset collected from five subjects, where each subject repeated each gesture five times. Therefore, the total number of test trials for each gesture was 25.

## 4. Results

Table 5 displays the evaluation metrics of the 5F-CV, including sensitivity, specificity, precision, and the F1 score. The F1 score, a metric particularly emphasized in our evaluation, offers a comprehensive assessment of the model’s accuracy by combining sensitivity and precision using their harmonic mean. Sensitivity (also known as recall) measures the model’s ability to correctly identify positive instances. It is calculated as the ratio of true positives (TP) to the sum of true positives and false negatives (FN):

(1) $${\mathrm{Sensitivity}}_{i}=\frac{{TP}_{i}}{{TP}_{i}+{FN}_{i}}$$

where *i* is number of class (*i* = 1, …, 10). Specificity, on the other hand, quantifies the model’s ability to avoid false positives. It is calculated as the ratio of true negatives (TN) to the sum of true negatives and false positives (FP):

(2) $${\mathrm{Specificity}}_{i}=\frac{{TN}_{i}}{{TN}_{i}+{FP}_{i}}$$

Precision, or positive predictive value, measures the model’s accuracy in predicting positive instances. It is calculated as the ratio of true positives to the sum of true positives and false positives:

(3) $${\mathrm{Precision}}_{i}=\frac{{TP}_{i}}{{TP}_{i}+{FP}_{i}}$$

The F1 score combines precision and sensitivity into a single metric, providing a balanced assessment of the model’s performance. It is calculated as the harmonic mean of precision and sensitivity:

(4) $$F_{1,i}=\frac{2\times {Precision}_{i}\times {Sensitivity}_{i}}{{Precision}_{i}+{Sensitivity}_{i}}$$

Total accuracy is calculated as the percentage of all gestures correctly identified across the ten classes. These metrics collectively offer a detailed understanding of the classifier’s performance, helping to evaluate its ability to accurately classify gestures within each class and across the entire dataset.

(5) $$\mathrm{Total}\;\mathrm{accuracy}=\frac{\sum_{i=1}^{10}\;({TP}_{i}+{TN}_{i})}{\sum_{i=1}^{10}{(TP}_{i}+{TN}_{i}+{FP}_{i}+{FN}_{i})}$$

In the context of each class, true positives (TP*_i_*) represent correctly identified gestures within that class, while true negatives (TN*_i_*) indicate gestures accurately recognized as not belonging to that class. False positives (FP*_i_*) refer to gestures incorrectly assigned to the class, and false negatives (FN*_i_*) are gestures in the class that were incorrectly classified into other classes. The total accuracy is determined by calculating the percentage of all gestures across the ten classes that were correctly identified. These metrics play a critical role in assessing the classifier’s performance, offering a detailed understanding of its ability to accurately classify gestures within each class and across the entire dataset.

The results presented in Table 5 demonstrate the impressive classification performance achieved. Notably, the sensitivity for 7 out of the 10 gestures exceeded 98%, and the overall accuracy reached 98.3%, showcasing the system’s exceptional ability to precisely recognize the diverse set of elbow gestures. The gestures with lower sensitivity values were gestures 1 and 3, which were predominantly misclassified with gestures 7 and 4, respectively. However, the sensitivity for these gestures still exceeds 95%. This higher rate of misclassification can be attributed to the inherent similarities between these two gestures, as gesture 4 involves bending at a 45-degree angle compared to gesture 3, and gesture 7 is the twisted version of gesture 1, making it challenging for the classification algorithm to reliably distinguish between the two. Despite this minor limitation, the overall results underscore the effectiveness of this approach in accurately identifying the various elbow movements, highlighting the potential of their technique for practical applications.

**Figure 4:**
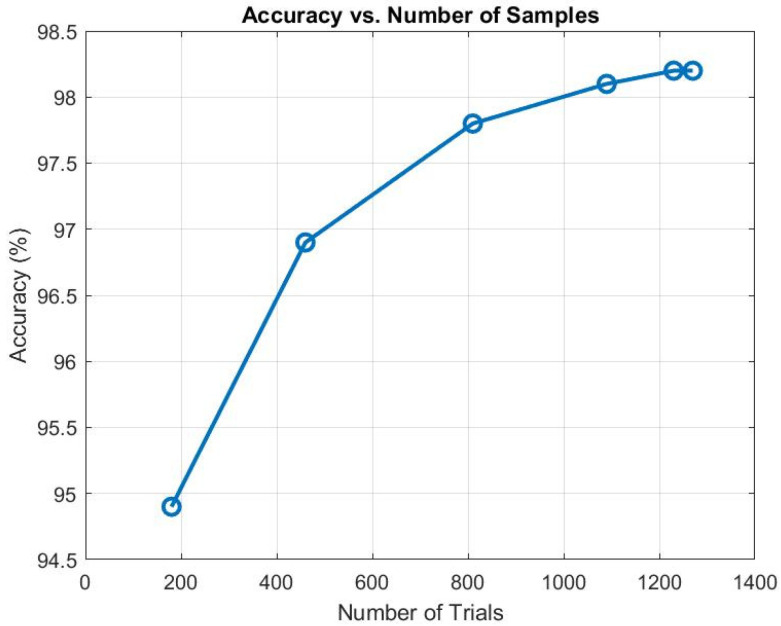
Accuracy as a function of the number of trials.

To evaluate the effect of the number of trials in overall classification accuracy, we evaluated the LOSO-CV performance from the case when augmenting data by adding only 140 trials from combinations of selecting 2 subjects from 8 subjects to the case when adding all 1230 trials from all combinations. Figure 4 shows the relationship between the number of trials and the accuracy of our model. As seen in Figure 4, with just 460 trials (adding combinations of selecting 2 and 3 subjects from 8 subjects), our model achieved an accuracy of 96.9%, indicating that even a relatively modest amount of augmented data can yield highly accurate results. As we increased the number of trials to 810, 1090, 1230, and 1270, we observed a corresponding improvement in performance, with the accuracy rising to 97.8%, 98.1%, 98.2%, and 98.2%, respectively. This incremental improvement underscores the benefit of additional data in enhancing model performance. However, it is important to note that beyond a certain point, specifically after 1230 trials, the accuracy gains plateaued, suggesting diminishing returns from further increasing the sample size. This observation aligns with the expectation that while more data generally lead to better model performance, there is a threshold beyond which additional trials contribute minimally to accuracy improvements.

**Figure 5:**
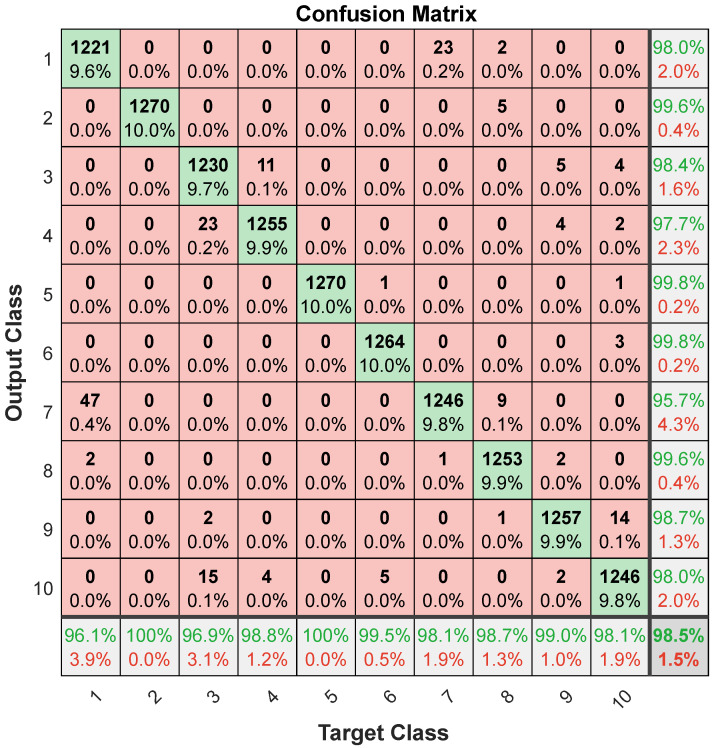
Aggregated confusion matrix for LOSO-CV. Column 11 shows the precision (highlighted in green) and the false positive (FP) rate (highlighted in red) for each class. Row 11 shows the sensitivity (highlighted in green) and the false negative (FN) rate (highlighted in red).

To further evaluate the generalizability of the model, in Figure 5, the confusion matrix for all 254 subjects (1270 trials) under LOSO-CV is depicted. Column 11 of the matrix shows the precision (highlighted in green) and the false positive (FP) rate (highlighted in red) for each class. Similarly, row 11 shows the sensitivity (also highlighted in green) and the false negative (FN) rate (highlighted in red). These metrics provide detailed insights into the classifier’s performance for each class, highlighting the system’s overall accuracy and reliability.

Table 6 presents the corresponding evaluation metrics (sensitivity, specificity, precision, and F1 score). The detailed performance metrics provided in Table 6 offer a comprehensive evaluation of the classifier’s capabilities across individual gesture classes. The reported overall accuracy is 98.5%, highlighting its robust and reliable classification capabilities. In Figure 5 and Table 6, the lowest sensitivity values again belong to gestures 1 and 3, which are still above 96%. The system’s ability to reliably classify a diverse range of elbow gestures, despite potential variability in factors like hand size, shape, and individual kinematic signatures, highlights its practical utility and versatility.

**Figure 6:**
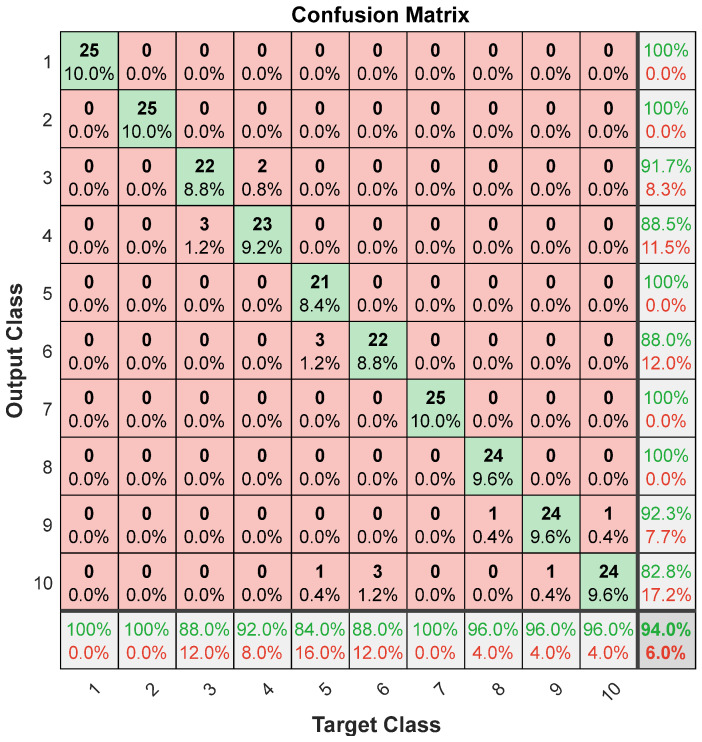
Test confusion matrix using 25 trials of the 5 test subjects. Column 11 shows the precision (highlighted in green) and the false positive (FP) rate (highlighted in red) for each class. Row 11 shows the sensitivity (highlighted in green) and the false negative (FN) rate (highlighted in red).

Finally, to test our model on unseen subjects, we tested the trained model on the new data collected from five subjects. The confusion matrix and the corresponding evaluation metrics for all 5 test subjects (25 trials per gesture) are illustrated in Figure 6 and Table 7. In Figure 6 and Table 7, the lowest performance is attributed to gesture 5, where only 4 out of 25 trials for this gesture are misclassified, which proves the high generalizability of the developed system one more time.

## 5. Discussion and Conclusions

This work presents a novel approach for elbow gesture recognition using an array of inductive sensors and machine learning techniques. The proposed solution addresses some of the key limitations of existing elbow gesture recognition systems. Compared to rigid MEMS sensors, the flexible inductive sensor array integrated into the wearable sleeve provides improved comfort and user experience. Unlike resistive sensors, the inductive approach is less susceptible to wear and tear, improving reliability over long-term use. Also, in contrast to capacitive sensing, the inductive sensors are less affected by external disturbances, further enhancing the system’s performance. Furthermore, the inductive sensor-based approach avoids the complex signal processing and precise sensor placement required by EMG-based systems. This simplifies the overall system design and makes it more accessible for a wider range of applications. Notably, in contrast to the previous wearable sensing works, there is no need to attach bulky or uncomfortable sensors to the elbow. This approach not only reduces the cost of the sensors significantly but also offers greater comfort for the user.

The current system prototype requires a wired connection to a PC to record the sensor data. Since the utilized microcontroller, NodeMCU Esp8266, offers Wi-Fi capability, the acquired responses can be transferred to a PC wirelessly for adding mobility to the system if further comfort of the user is needed in the future. Moreover, the current system uses an array of four inductive sensors sewn onto the flexible sleeve. While this sensor array was able to capture a range of 10 elbow gestures with high accuracy, future work could explore incorporating a larger number of optimized sensors on the sleeve. This could enable the detection of more complex and nuanced elbow movements, as well as potentially allow the system to be extended to monitor motions of other joints, such as the shoulder or even lower limb movements.

It is worth noting that proper fit is crucial for the sensor array to accurately capture the intended elbow movements. Future iterations of the design could explore techniques to enable easy size adjustments or even personalized fabrication of the sleeves.

The rigorous testing conducted on 8 subjects, with data augmentation leveraging the dataset to 1270 trials per gesture, enabled the system to achieve remarkable evaluation accuracy of 98.3% and 98.5% using 5F-CV and LOSO-CV, respectively, as well as a high accuracy of 94% based on 25 trials per gesture from 5 subjects. The combination of the flexible inductive sensor array and the robust RF MLA appears to be a highly effective solution for intuitive human–machine interaction applications. The inductive sensors can capture a wide range of elbow movements, while the machine learning model can reliably distinguish between 10 different gestures.

The presence of a resting position does not imply that only elbow gestures starting from the resting position can be recognized. In practice, calibration is typically performed by starting from the resting position initially and only once. Then, the user can perform various gestures, and recognition of them can be implemented without the need to repeat the resting gesture anymore. However, in our study, we initiated data collection from the resting position as a standard procedure for each gesture and sample taken from participants to ensure the smoothness of data collection and to align with our data-processing method for the system.

Overall, this work presents a promising and practical solution for elbow gesture recognition, with the potential to enable more intuitive human–machine interactions in a variety of applications, from rehabilitation and assistive technologies to gaming, robotics control, and the animation industry.

## Figures and Tables

**Table 1:** Parameters used for the single-layer rectangular coil model for four sensors.

Sensor Parameter	Sensor 1	Sensor 2	Sensor 3	Sensor 4
Width (mm)	40	50	60	50
Length (mm)	60	70	70	110
Gap between turns (mm)	2	2	2	2
Number of turns	4	8	6	5

**Table 2:** Resonant frequency alternations (mean ± std) in MHz for each sensor and all gestures for a single user.

Gesture	Sensor 1	Sensor 2	Sensor 3	Sensor 4
1	0.02118 ± 0.00228	0.01694 ± 0.00078	0.01186 ± 0.00268	0.01882 ± 0.00628
2	0.01079 ± 0.00173	0.00913 ± 0.00184	0.00633 ± 0.00065	0.00507 ± 0.00100
3	0.00244 ± 0.00037	0.01116 ± 0.00132	0.00193 ± 0.00058	0.00372 ± 0.00186
4	0.00243 ± 0.00029	0.00523 ± 0.00041	0.00265 ± 0.00037	0.00230 ± 0.00064
5	0.00009 ± 0.00009	0.00013 ± 0.00005	0.00014 ± 0.00011	0.00013 ± 0.00010
6	0.00107 ± 0.00095	0.00308 ± 0.00053	0.00057 ± 0.00018	0.00514 ± 0.00083
7	0.02483 ± 0.00251	0.03823 ± 0.00343	0.01911 ± 0.00146	0.00572 ± 0.00244
8	0.00607 ± 0.00120	0.01201 ± 0.00162	0.00490 ± 0.00098	0.01213 ± 0.00142
9	0.00469 ± 0.00052	0.00793 ± 0.00135	0.00155 ± 0.00106	0.00476 ± 0.00084
10	0.00460 ± 0.00047	0.00441 ± 0.00123	0.00121 ± 0.00050	0.00769 ± 0.00124

**Table 3:** Resonant frequency alternations (mean ± std) in MHz for each sensor, for all gestures, and for all users.

Gesture	Sensor 1	Sensor 2	Sensor 3	Sensor 4
1	0.02464 ± 0.00322	0.02463 ± 0.00727	0.01187 ± 0.00274	0.00850 ± 0.00743
2	0.00938 ± 0.00469	0.01243 ± 0.00461	0.00381 ± 0.00198	0.00339 ± 0.00188
3	0.00163 ± 0.00075	0.01148 ± 0.00431	0.00346 ± 0.00151	0.00471 ± 0.00267
4	0.00190 ± 0.00099	0.00740 ± 0.00252	0.00243 ± 0.00194	0.00252 ± 0.00092
5	0.00010 ± 0.00008	0.00009 ± 0.00006	0.00007 ± 0.00003	0.00005 ± 0.00004
6	0.00162 ± 0.00134	0.00529 ± 0.00136	0.00053 ± 0.00035	0.00532 ± 0.00281
7	0.02788 ± 0.00351	0.03504 ± 0.00542	0.01585 ± 0.00257	0.00653 ± 0.00316
8	0.00645 ± 0.00226	0.01016 ± 0.00318	0.00273 ± 0.00191	0.00995 ± 0.00262
9	0.00382 ± 0.00171	0.00753 ± 0.00165	0.00466 ± 0.00129	0.00792 ± 0.00284
10	0.00981 ± 0.00207	0.00440 ± 0.00197	0.00251 ± 0.00098	0.00747 ± 0.00276

**Table 4:** Sensors’ sensitivities (Hz/degree) for various postures.

Gesture	Sensor 1	Sensor 2	Sensor 3	Sensor 4
Front bending	235.33	188.22	131.77	209.11
Side bending	27.11	124	21.44	41.33
Twisting	11.88	34.22	63.33	57.11

**Table 5:** Evaluation performance of RF-Classifier using 5F-CV for 4 sensors.

Classes	Sensitivity (%) (Mean ± Std)	Specificity (%) (Mean ± Std)	Precision (%) (Mean ± Std)	F1 (%) (Mean ± Std)	Total Accuracy (%) (Mean ± Std)
1	95.89 ± 0.27	99.75 ± 0.02	97.67 ± 0.23	96.77 ± 0.14	
2	99.95 ± 0.06	99.96 ± 0.01	99.65 ± 0.07	99.8 ± 0.041	
3	96.39 ± 0.34	99.71 ± 0.04	97.39 ± 0.41	96.89 ± 0.21	
4	98.06 ± 0.36	99.64 ± 0.05	96.82 ± 0.49	97.43 ± 0.26	
5	99.99 ± 0.02	99.99 ± 0	99.92 ± 0	99.96 ± 0.01	98.30 ± 0.08
6	99.50 ± 0.08	99.97 ± 0.01	99.71 ± 0.08	99.61 ± 0.06	
7	97.76 ± 0.24	99.52 ± 0.03	95.73 ± 0.25	96.73 ± 0.13	
8	99.02 ± 0.15	99.94 ± 0.01	99.49 ± 0.12	99.25 ± 0.07	
9	98.34 ± 0.23	99.84 ± 0.03	98.53 ± 0.31	98.43 ± 0.18	
10	98.06 ± 0.32	99.79 ± 0.02	98.13 ± 0.23	98.09 ± 0.19	

**Table 6:** Evaluation performance of RF-Classifier using LOSO-CV for 4 sensors.

Classes	Sensitivity (%)	Specificity (%)	Precision (%)	F1 (%)	Total Accuracy (%)
1	96.14	99.78	97.99	97.06	
2	100.00	99.96	99.61	99.80	
3	96.85	99.83	98.40	97.62	
4	98.82	99.75	97.74	98.28	
5	100.00	99.98	99.84	99.92	
6	99.53	99.97	99.76	99.65	98.51
7	98.11	99.51	95.70	96.89	
8	98.66	99.96	99.60	99.13	
9	98.98	99.85	98.67	98.82	
10	98.11	99.77	97.96	98.03	

**Table 7:** Testing performance of RF-Classifier using 25 trials of the 5 test subjects.

Classes	Sensitivity (%)	Specificity (%)	Precision (%)	F1 (%)	Total Accuracy (%)
1	100.00	100.00	100.00	100.00	
2	100.00	100.00	100.00	100.00	
3	88.00	99.11	91.67	89.80	
4	92.00	98.67	88.46	90.20	
5	84.00	100.00	100.00	91.30	
6	88.00	98.6667	88.00	88.00	94.00
7	100.00	100.00	100.00	100.00	
8	96.00	100.00	100.00	97.96	
9	96.00	99.11	92.31	94.12	
10	96.00	97.78	82.76	88.89	

## Data Availability

The original contributions presented in the study are included in the article; further inquiries can be directed to the corresponding author.
